# Proton pump rhodopsins for optogenetic manipulation of biological activities and beyond

**DOI:** 10.1016/j.jbc.2026.113318

**Published:** 2026-07-09

**Authors:** Keiichi Kojima, Miyu Inokuchi, Yuki Sudo

**Affiliations:** 1 Faculty of Medicine, Dentistry and Pharmaceutical Sciences, Okayama University, Okayama, Japan; 2 Graduate School of Medicine, Dentistry and Pharmaceutical Sciences, Okayama University, Okayama, Japan

**Keywords:** H^+^ pump, optogenetics, pH, proton motive force, retinal, rhodopsin

## Abstract

Water (H_2_O), the principal component of living organisms including humans, dissociates into H^+^ and OH^-^ in aqueous environments, and the resulting H^+^ concentration determines both cellular pH and the proton motive force (PMF) across cellular membranes. These physicochemical parameters are fundamental regulators of a wide range of biological processes. Optogenetics enables the manipulation of biological and cellular functions using light, typically through the ectopic expression of microbial rhodopsins as photoreceptive proteins in target cells or organs. This review provides a comprehensive overview of optogenetic studies employing H^+^ pump rhodopsins in diverse biological systems and highlights their growing relevance to neuroscience, cell biology, bioengineering, and therapeutic research. Notably, optical control of H^+^ concentration allows the precise modulation of neural and non-neural activities in animals and bacteria, highlighting the broad applicability and significant potential of H^+^ pump rhodopsins for optogenetics. Based on previous and current studies, we discuss how further expansion of the molecular toolkit of H^+^ pump rhodopsins could enable increasingly fine-tuned manipulation of intracellular pH dynamics and PMF for probing cellular physiology and designing next-generation therapeutic strategies and consider emerging directions that extend beyond classical optogenetics toward the optical control of bioenergetic and chemical processes.

Living organisms consist of cells containing biological components such as nucleotides, proteins, and lipids. Notably, these components are surrounded by water (H_2_O) molecules (55–75% in human body) (1). The intracellular spaces of unicellular and multicellular organisms, as well as the extracellular spaces of multicellular organisms, are generally filled with aqueous solutions in which H_2_O dissociates into H^+^ (proton) and OH^-^ (Fig. 1*A*). pH, defined as −log[H^+^], serves as an index of H^+^ concentration ([H^+^]). This logarithmic relationship means that a difference of one pH unit corresponds to a tenfold change in H^+^ concentration. Because pH is one of the critical regulators of biological reactions, living organisms have evolved regulatory mechanisms that precisely control both intracellular and extracellular pH to maintain homeostasis, in addition to the conventional buffering effects such as phosphate ions in these environments (2, 3). For example, in humans, blood pH is tightly regulated at approximately 7.35 to 7.45 (Fig. 1*A*) (4). In mammalian cells, the cytosol pH is typically ∼7.2 (range 7.0–7.6), whereas individual cellular organelles exhibit characteristic pH values. For example, the pH is ∼7.2 in the nucleus, 7.1 to 7.4 in the endoplasmic reticulum, 6.0 to 6.7 in the Golgi apparatus, 6.0 to 6.5 in early endosomes, <6.0 in late endosomes, <5.0 in lysosomes, 7.8 to 8.0 in the mitochondrial matrix (compared with ∼6.8 in the intermembrane space), and ∼7.0 in peroxisomes (Fig. 1*B*) (3, 5). Neutralophilic bacteria can grow over a broad range of external pH values (∼5.5–9.0) yet generally maintain their cytoplasmic pH within a narrow range of ∼7.5 to 7.7 (Fig. 1*C*) (6).

**Figure 1:**
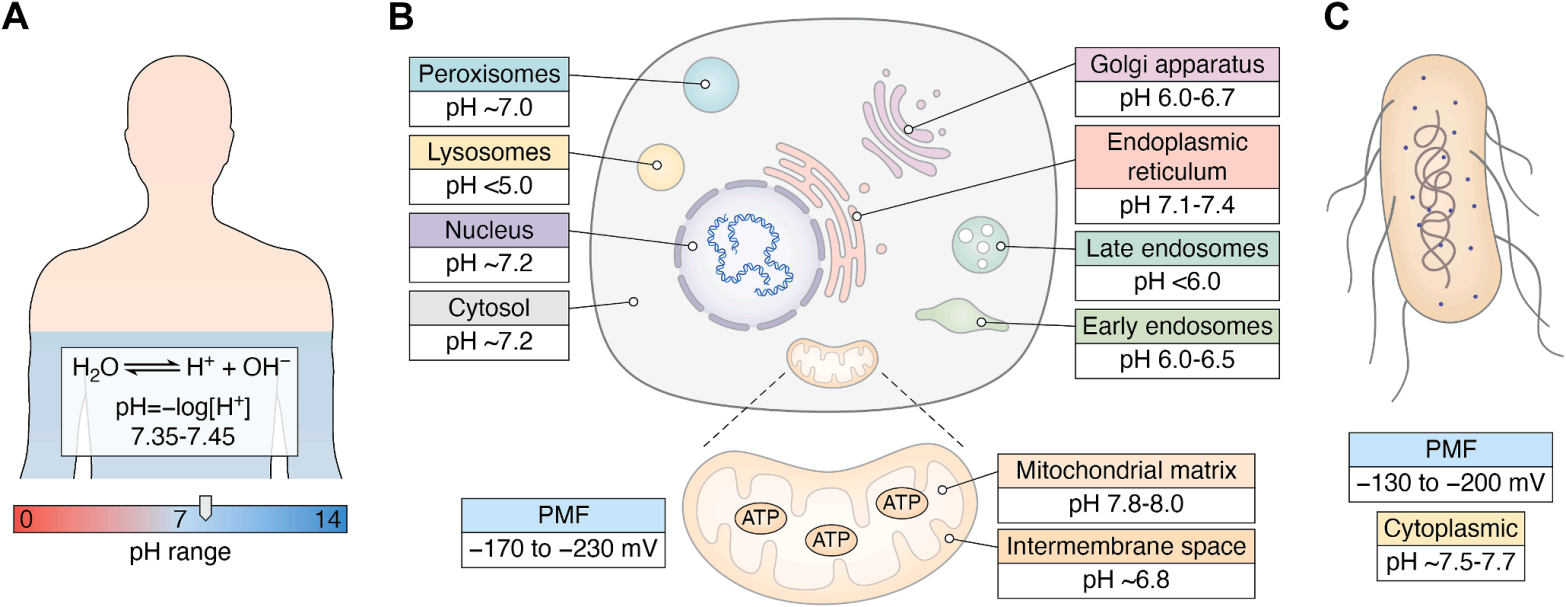
**Biological significance of H^+^ concentrations as determinants of pH and proton motive force (PMF).***A*, the main constituent of the human body is water (H_2_O). H_2_O dissociates into H^+^ and OH^-^, and blood pH is tightly regulated at approximately 7.35 to 7.45. *B*, in mammalian cells, the cytosol typically has a pH of ∼7.2, while cellular organelles have characteristic pH values. The PMF (−170 to −230 mV) across the mitochondrial inner membrane, generated by respiration, drives ATP synthesis. *C*, the cytoplasmic pH of most bacteria is also maintained around 7.5 to 7.7. Bacteria generally possess similar biochemical mechanisms that utilize the PMF (−130 to −200 mV). The PMF regulates bacterial swimming by driving the rotation of the flagellar motor.

pH also contributes to the generation of the proton motive force (PMF) across cellular membranes, because the PMF is defined as [PMF= −(2.3*RT*/*F*) × ΔpH + Δψ], where *R*, *T*, *F* and ψ represent gas constant, temperature, Faraday constant and membrane voltage, respectively. Thus, it not only contributes to membrane potential formation but also serves as an energy source for various PMF-driven transporters and for powering rotation of the bacterial flagellar motor (flagellar motor rotation), a membrane-embedded nanomachine that drives bacterial swimming motility (Fig. 1*C*).

In eukaryotic cells, the PMF (−170 to −230 mV) across the mitochondrial inner membrane, generated by respiration, drives ATP synthesis (7) (Fig. 1*B*). Prokaryotes possess similar PMF-dependent mechanisms (−130 to −200 mV) (8), consistent with the notion that eukaryotic mitochondria originated from symbiotic bacteria (Fig. 1*C*). Thus, H^+^ concentration is a key factor for sustaining biological processes in living organisms by regulating both intracellular and extracellular pH as well as the PMF.

Optogenetics is a method for controlling biological and cellular responses using light, in which photoreactive proteins are ectopically expressed in target cells or organs (9, 10). In optogenetics, rhodopsins, a family of photoreactive membrane proteins that contain retinal, the chromophore essential for rhodopsin function, serve as the primary actuators that initiate cellular responses upon light illumination (Fig. 2*A*). Rhodopsins bind retinal *via* a Schiff base linkage to a conserved lysine residue located in the seventh transmembrane helix and are phylogenetically divided into two classes, animal and microbial rhodopsins (11, 12). Animal rhodopsins generally function as G protein-coupled receptors (GPCRs) that activate heterotrimeric G proteins to trigger diverse intracellular signaling pathways (13, 14, 15), whereas microbial rhodopsins primarily act as ion transporters (pumps and channels) and regulators of kinases and enzymes, driving energy production and photo-responses such as phototaxis in microorganisms (12, 16, 17). Microbial rhodopsins typically bind all-*trans* retinal in the dark state. After the photoisomerization from all-*trans* to 13-*cis* retinal, rhodopsins sequentially form several spectroscopically distinct photointermediates (K, L, M, N, and O), after which the protein returns to the initial dark state accompanied by retinal reisomerization. This cyclic reaction, known as the photocycle, underlies the molecular functions of microbial rhodopsins (Fig. 2*B*). The first microbial rhodopsin, bacteriorhodopsin (HsBR), was identified in 1971 from the haloarchaeon *Halobacterium salinarum* as an outward H^+^ pump that transports protons from the cytoplasmic side to the extracellular side, generating a PMF across the cell membrane (18). The resulting PMF is utilized by H^+^-ATPase to produce ATP as an energy source for the archaea. Recent advances in metagenomic analyses of microorganisms have revealed diverse microbial rhodopsins including inward Cl^−^ pumps, outward Na^+^ pumps, inward H^+^ pumps, cation channels, and anion channels, in archaea, bacteria, eukaryotic microorganisms, and viruses (Fig. 2*C*) (16). After the first demonstration of optogenetics, microbial rhodopsins were primarily employed to optically control the membrane potential of neurons in specific brain regions, thereby regulating neural activity both *in vitro* and *in vivo*. However, due to the extensive molecular diversity of microbial rhodopsins, as illustrated in Figure 2*C*, their diverse functional properties may also be applied to the optical control of non-neural physiological processes.

**Figure 2:**
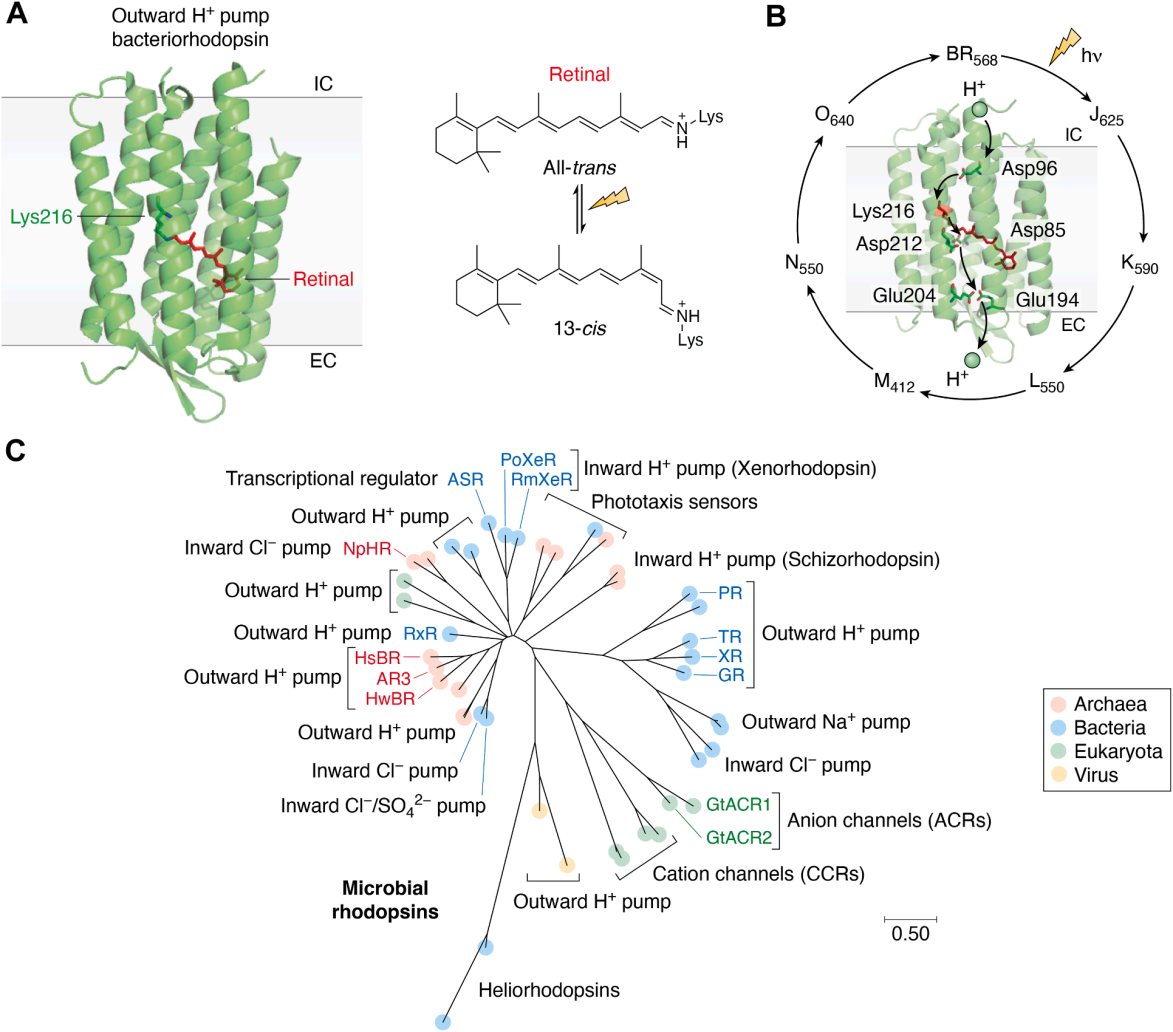
**Molecular basis and diversity of microbial rhodopsins.***A*, crystal structures of the outward proton pump rhodopsin bacteriorhodopsin (HsBR) (PDB ID: 1C3W). The retinal chromophore (*colored red*) is covalently bound to a conserved lysine residue of the apoprotein opsin *via* a Schiff base linkage, and its photoisomerization from the all-trans to the 13-*cis* form is triggered. Retinal and Lys216 are highlighted in the structure. *B*, schematic representation of the photoreaction process of HsBR. The absorption maxima of the intermediates and the time constants of each transition are shown. The proton-transport pathway of HsBR is also depicted. Key residues involved in proton transport (Asp85, Asp96, Glu194, Glu204, and Asp212), retinal, and Lys216 are highlighted in the structure. IC and EC denote the intracellular and extracellular sides, respectively. *C*, phylogenetic tree of microbial rhodopsins. Rhodopsins from archaea, bacteria, eukarya, and viruses are indicated by filled circles colored *red*, *blue*, *green*, and *brown*, respectively. The scale bar represents the average number of amino acid substitutions per site. The phylogenetic tree was inferred using the maximum likelihood method of MEGA 12 software.

In this review, we focus on H^+^ pump rhodopsins, which have the longest research history and are the most conventional members among the diverse microbial rhodopsins. Notably, outward H^+^ pump rhodopsins have been identified across all biological domains of life (*i*.*e*., archaea, bacteria, and eukaryotic microorganisms) as well as in viruses (Fig. 2*C*) (16), suggesting the fundamental importance of H^+^ homeostasis for biological function in various organisms. Moreover, inward H^+^ pump rhodopsins, which transport protons from the extracellular side to the cytoplasmic side, have been discovered in archaea and bacteria (19, 20). The availability of both outward and inward H^+^ pumps now enables bidirectional optical control of cellular pH and PMF. This review provides a comprehensive overview of representative optogenetic studies employing H^+^ pump rhodopsins to regulate diverse biological activities and highlights their high potential to drive a broad range of chemical reactions beyond optogenetics (Table 1).

**Table 1:** Optogenetic applications of H^+^ pump rhodopsins

Microbial rhodopsin	Molecular function	Target cell, compartment	Application	Ref
AR3 and variants	Outward H^+^ pump	Neurons	Neural silencing	(25, 28, 29, 30)
		Bacteria	Flagellar motor rotation	(51)
		Synaptic vesicles	Neurotransmitter accumulation	(52)
		Lysosomes	Alterations in hydrolase activity	(52)
		Mitochondria	Suppression of cell death, ATP synthesis, lifespan extension	(55, 56, 57, 58)
		Mammalian cells, cancers	Apoptosis	(66, 67)
		Mammalian cells, neurons	Voltage imaging	(73, 74, 75, 76, 77, 78, 79)
Arch-T	Outward H^+^ pump	Neurons	Neural silencing	(27, 30)
		Lysosomes	Alterations in hydrolase activity	(53)
		Mammalian cells	Plasma membrane ruffling	(63)
PR	Outward H^+^ pump	Bacteria	ATP synthesis	(40)
		Bacteria	Flagellar motor rotation	(49, 50)
		Bacteria	Voltage imaging	(72)
GR	Outward H^+^ pump	Bacteria	ATP synthesis, growth, CO_2_ fixation	(41, 42, 44, 45)
dR	Outward H^+^ pump	Bacteria	ATP synthesis, carbon metabolism	(43)
		Bacteria	Efflux of toxic compounds	(46)
D217E mutant of ASR	Inward H^+^ pump	Endosomes	Inhibition of endocytosis	(54)
RmXeR	Inward H^+^ pump	Bacteria	Flagellar motor rotation	(51)
		Mammalian cells	Inhibition of apoptosis	(66)
		Liposomes in mammalian cells	Substance delivery	(69)

## Classical optogenetics of neural activities using microbial rhodopsins

**Figure 3:**
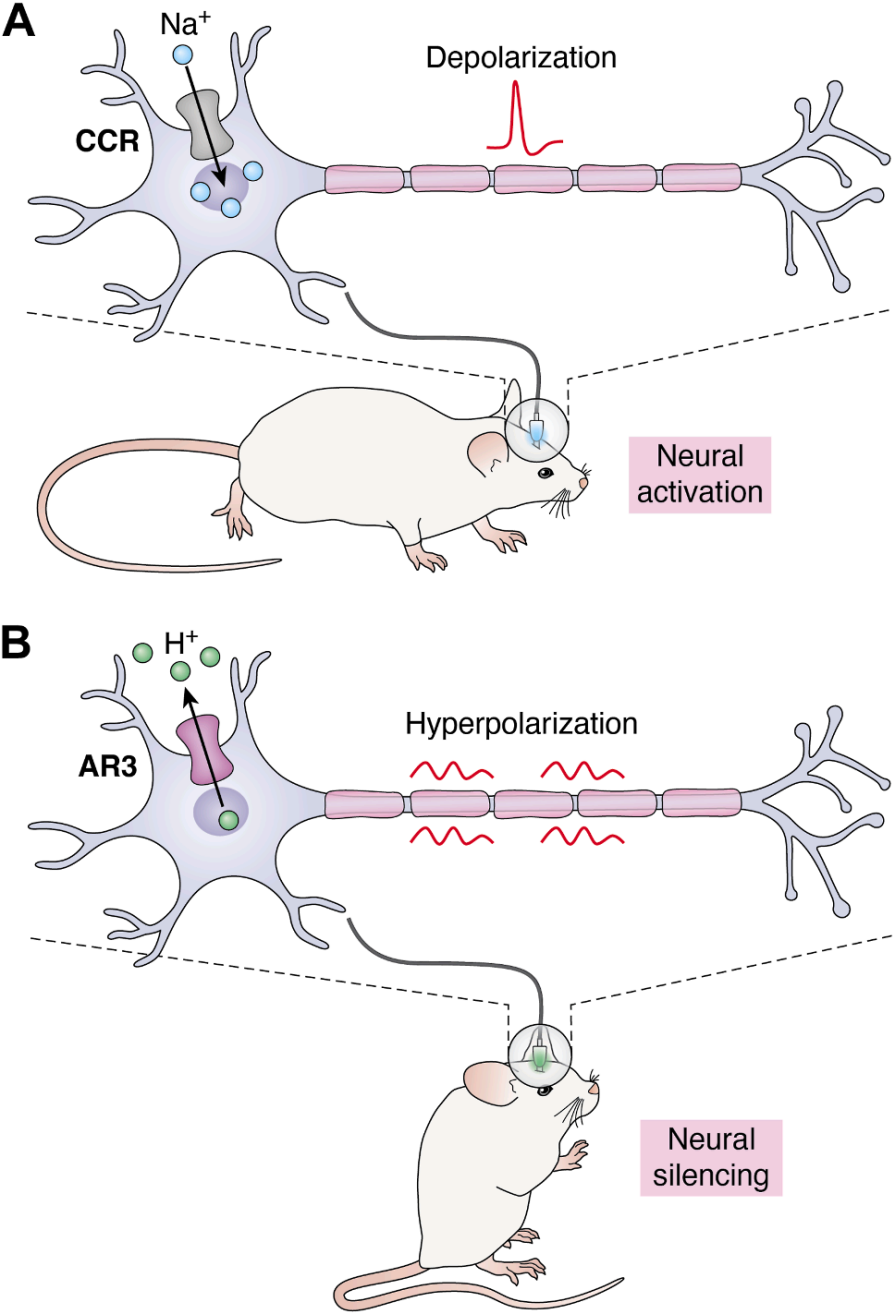
**Optical control and imaging of neural activities with H^+^ pump rhodopsins.***A*, schematic of neural activation using cation channelrhodopsins (CCRs). Light irradiation induces neuronal depolarization through inward cation transport (mainly Na^+^), leading to neural activation. *B*, schematic of neural inhibition using outward H^+^ pump rhodopsins, such as AR3 and Arch-T. Light irradiation induces hyperpolarization through outward H^+^ transport, thereby causing neural silencing.

In 2005, Dr Deisseroth’s group and several other groups independently ectopically expressed a cation channelrhodopsin (CCR), a light-gated cation channel, identified from the alga *Chlamydomonas reinhardtii*, in animal neurons (21, 22, 23, 24). They demonstrated that light irradiation induces neuronal depolarization through inward cation transport (mainly Na^+^), leading to neural activation (Fig. 3*A*). In contrast, it has been shown that an inward Cl^-^ pump halorhodopsin and outward H^+^ pump rhodopsins induce hyperpolarization through inward Cl^−^ and outward H^+^ transport, respectively, thereby causing neural inhibition (Fig. 3*B*) (25, 26). Among outward H^+^ pump rhodopsins, Archaearhodopsin-3 (AR3 or Arch) from the archaeon *Halorubrum sodomense* and Archaerhodopsin-T (Arch-T) from the archaeon *Halorubrum trapanicum* have been used most widely because they show higher protein expression levels, greater membrane localization, and more robust photocurrents in mammalian and neuronal cells compared with other outward H^+^ pump rhodopsins (25, 27, 28, 29, 30). However, because H^+^ pump rhodopsins actively transport protons, they can induce secondary ion fluxes, including inward cation and outward anion movements, which contribute to membrane hyperpolarization. These secondary ionic effects can limit the efficiency and precision of neural inhibition mediated by H^+^ pump rhodopsins. In 2015, natural anion channel rhodopsins (ACRs), light-gated anion channels identified from the cryptophyte alga *Guillardia theta* (GtACR1 and GtACR2), were shown to induce robust hyperpolarization and neural inhibition (31). To date, a wide variety of CCRs, including potassium-selective channel rhodopsins (KCRs), as well as ACRs with diverse spectral sensitivities ranging from violet to red and distinct channel opening and closing kinetics, have been identified from nature and further engineered by mutagenesis (9, 16, 32, 33). These microbial rhodopsins have been extensively employed to optically manipulate neuronal membrane potentials. Consequently, microbial rhodopsins have become the primary optogenetic tools for regulating neural activity mainly in neuroscience.

## Optical control of bacterial activities using H^+^ pump rhodopsins

**Figure 4:**
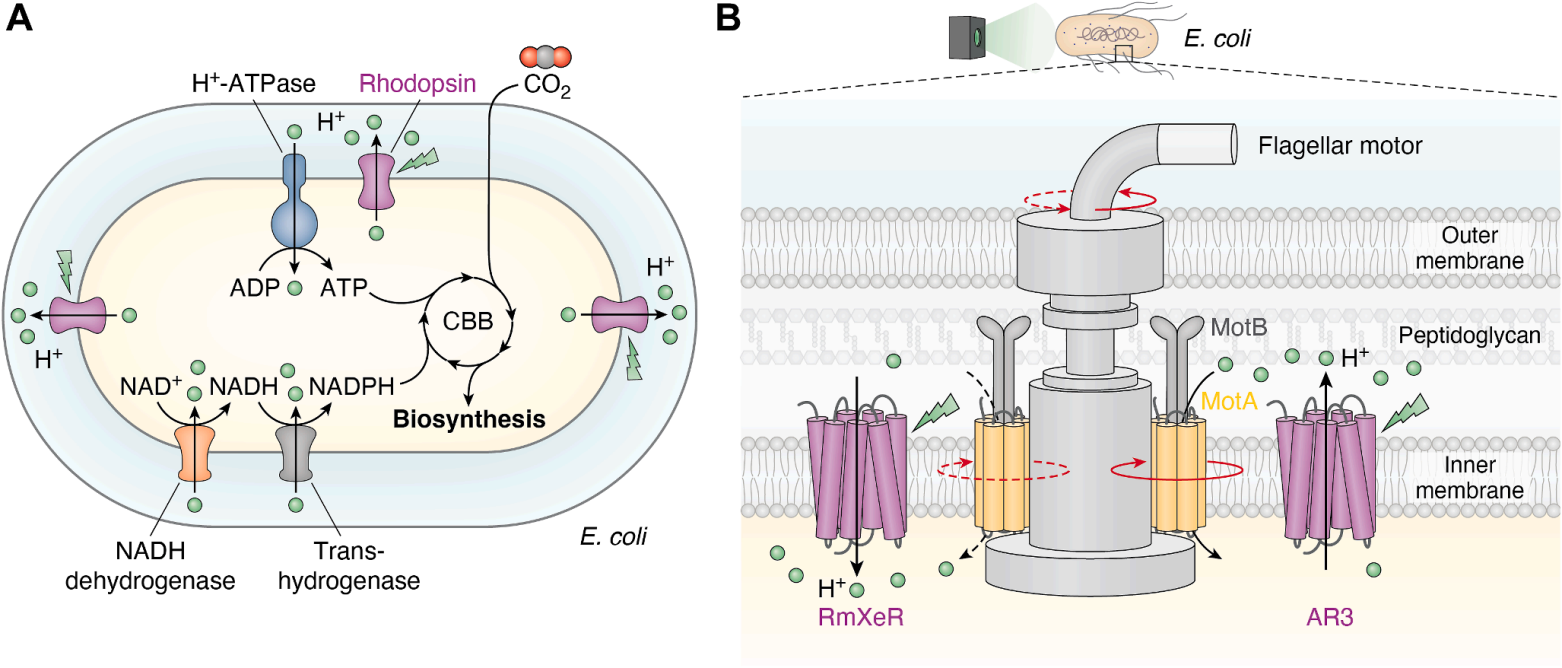
**Optical control of ATP production and bacterial motility using H^+^ pump rhodopsins.***A*, schematic of light-driven ATP production and CO_2_ fixation using outward H^+^-pump rhodopsins. Light irradiation generates PMF through outward H^+^ transport by rhodopsins, which in turn activates H^+^-ATPase, NADH dehydrogenase, and transhydrogenase, resulting in ATP, NADP^+^, and NADPH production, respectively, in bacterial cells. The light-generated ATP and NADPH serve as energy and reducing power for the Calvin–Benson–Bassham (CBB) cycle, thereby driving CO_2_ fixation and supporting biosynthesis. *B*, schematic of light-controlled flagellar motor rotation using H^+^ pump rhodopsins. The flagellar motor rotation is driven by the inward transport of H^+^ through a H^+^ channel (MotA−MotB complex) across the inner membrane. Light irradiation either generates or dissipates PMF through outward or inward H^+^ transport by AR3 and RmXeR, respectively, resulting in acceleration or deceleration of flagellar motor rotation in bacterial cells.

In halophilic archaea, H^+^ pump rhodopsins are expressed in the cell membrane and generate a PMF, which is utilized by H^+^-ATPase to synthesize ATP in a manner analogous to photosynthesis (18). In 1974, Racker and Stoeckenius demonstrated that the PMF generated by light-activated HsBR could drive ATP synthesis in reconstituted vesicles (34). This seminal finding suggested that H^+^ pump rhodopsins could be exploited to optically control PMF-dependent bacterial activities, including ATP synthesis, cellular growth, and flagellar motor function in microorganisms (Fig. 4). However, HsBR consistently failed to form photoactive holoproteins retaining the chromophore retinal when heterologously expressed in bacterial systems, which severely limited its application for artificial, light-based control of bacterial PMF. This limitation was overcome by the discovery of diverse microbial rhodopsins in the 2000s, which was initiated by the identification of the outward H^+^ pump rhodopsin, proteorhodopsin (PR), in γ-proteobacteria. Béjà *et al*. demonstrated that PR enables bacteria to convert solar energy into PMF *via* outward H^+^ transport across the cell membrane, and that the light-generated PMF drives ATP synthesis (35, 36). Indeed, the outward H^+^ pump rhodopsins have been identified from diverse microorganisms, such as halophilic archaea, proteobacteria, bacteroidetes, bdellovibrionota, cyanobacteria, thermophilic bacteria, algae, fungi, and viruses (Fig. 2*C*) (11, 12, 16, 17). In parallel, inward H^+^ pump rhodopsins were also discovered from bacteria and archaea. A phylogenetically distinctive rhodopsin clade, xenorhodopsin (XeR), from the bacterium *Parvularcula oceani* (PoXeR) was identified as the first natural inward H^+^ pump rhodopsin, followed by additional members of the xenorhodopsin clade, such as *Rubricoccus marinus* xenorhodopsin and *Nanosalina* xenorhodopsin (RmXeR and NsXeR, respectively) (19, 37, 38). Moreover, a phylogenetically distinct clade identified through metagenomic analyses has been named schizorhodopsin (SzR) (20, 39). Importantly, many of these rhodopsins can be heterologously expressed as photoactive holoproteins in *Escherichia coli*. This technological advance enabled systematic applications of microbial rhodopsins for the light-based control of bacterial physiology (Fig. 4). Several outward H^+^ pump rhodopsins, including PR, *Gloeobacter* rhodopsin (GR) from *Gloeobacter violaceus*, and delta rhodopsin (dR) from *Haloterrigena turkmenica*, have been utilized to drive light-dependent ATP synthesis, phototrophic growth, and metabolic engineering applications in *E. coli*. These applications include CO_2_ fixation through the Calvin–Benson–Bassham (CBB) cycle powered by light-generated ATP and NADPH, as well as the production of valuable compounds such as glutathione (40, 41, 42, 43, 44, 45) (Fig. 4*A*). In addition, co-expression of dR with EmrE, a small multidrug resistance (SMR) family transporter that utilizes PMF to export toxic compounds, enabled light-driven efflux of the substrate ethidium in *E. coli* cells (46).

Bacteria use flagella to migrate toward nutrients or favorable environments and to escape from harmful substances or unfavorable conditions, a behavior known as taxis (47, 48). The driving force for flagellar rotation is generated by ion flux through a membrane-embedded stator complex located at the flagellar basal body. In *E. coli* and *Salmonella*, the stator specifically conducts H^+^, whereas in *Vibrio* species it conducts Na^+^. Walter *et al*. heterologously expressed PR in *E. coli* cells and demonstrated that PR generates PMF and drives the flagellar motor, causing cells to swim when illuminated (49). Building on this concept, another group quantitatively analyzed the rotation rate of the motor and revealed the relationship between PMF and the diffusion dynamics of stator units away from the flagellar motor (50). Furthermore, we introduced not only outward but also inward H^+^ pump rhodopsins (AR3 and RmXeR, respectively) into *E. coli* cells and demonstrated bidirectional optical control of PMF, ranging from −140 to +146 mV, accompanied by corresponding increases or decreases in the rotation rate of the flagellar motor (51) (Fig. 4*B*). Thus, these studies clearly demonstrate that rhodopsin-driven, light-generated PMF can regulate various bacterial activities and enable the construction of an artificial photosynthetic system linked to carbon metabolism in engineered bacteria, which may be applied to biofuel production.

## Optical control of animal cell activities using H^+^ pump rhodopsins

**Figure 5:**
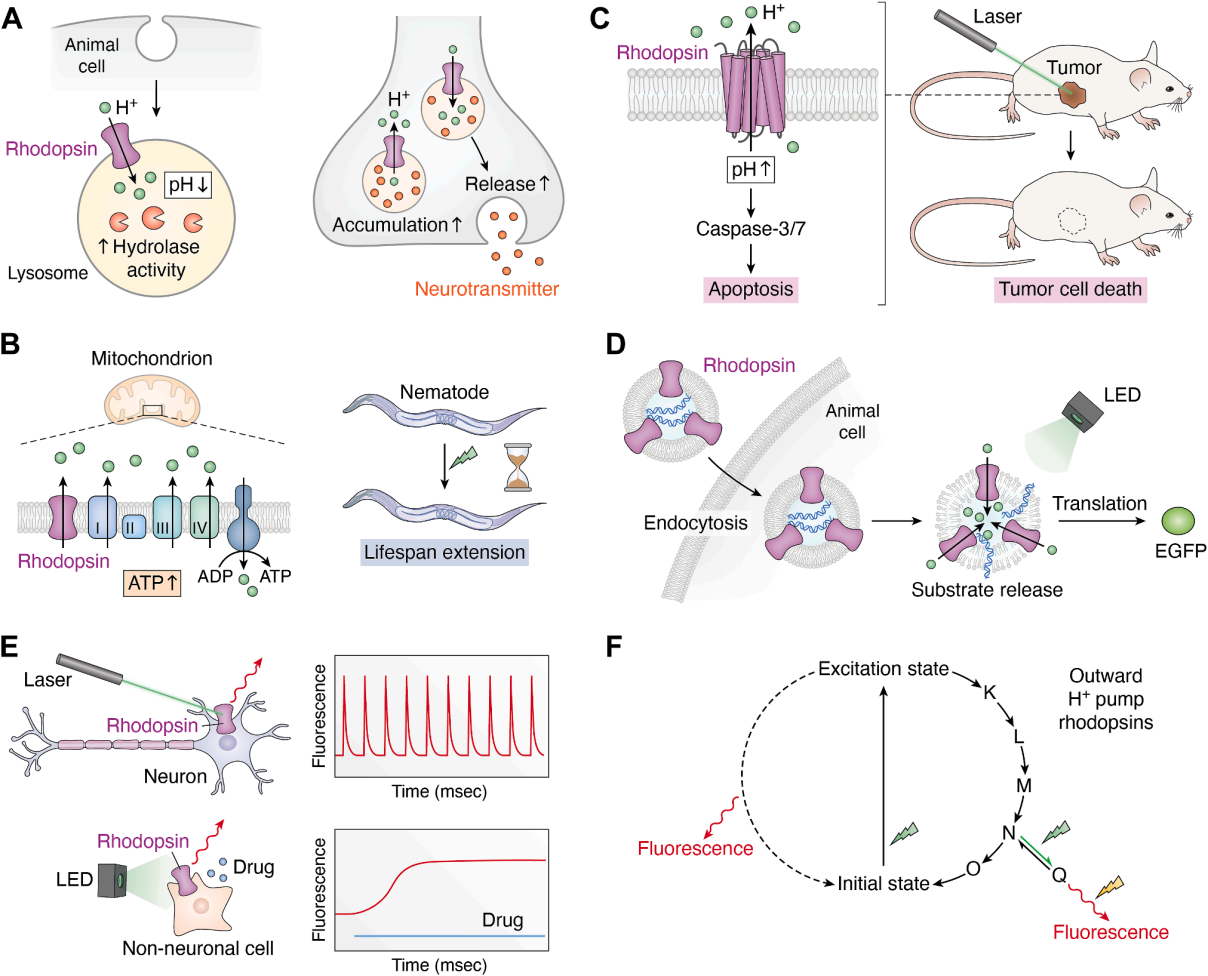
**Optical control and imaging of animal cell activities using H^+^ pump rhodopsins.***A*, schematic of light-controlled molecular functions in synaptic vesicles and lysosomes using H^+^ pump rhodopsins. Light irradiation modulates luminal pH through outward or inward H^+^ transport by rhodopsins, leading to increased neurotransmitter accumulation or release in synaptic vesicles and altered hydrolase activity in lysosomes of animal cells. *B*, schematic of light-controlled mitochondrial functions using outward H^+^ pump rhodopsins. Light irradiation promotes PMF generation across the mitochondrial inner membrane *via* outward H^+^ transport by rhodopsins, resulting in suppression of respiration-inhibition–induced cell death, enhanced ATP synthesis, and lifespan extension of the nematode. *C*, schematic of light-induced cell death mediated by outward H^+^ pump rhodopsins. Light irradiation induces intracellular alkalization through outward H^+^ transport by rhodopsins, thereby triggering mitochondria-mediated apoptosis in tumor cells. *D*, schematic of light-disruptive, rhodopsin-based liposomes that release encapsulated substrates upon illumination. Light irradiation triggers the release of encapsulated molecules, including mRNA encoding enhanced *green* fluorescent protein, into the cytosol, where the mRNA is translated and the encoded protein is expressed. IC and EC denote the intracellular and extracellular sides, respectively. *E*, schematic illustration of optical imaging of neural activities using outward H^+^ pump rhodopsins. These rhodopsins exhibit voltage-dependent near-infrared fluorescence changes, enabling detection of membrane voltage dynamics. Fluorescence changes can be monitored with high temporal resolution (∼100 ms) for real-time imaging of neural activities under intense laser illumination. In contrast, under low-intensity LED illumination, fluorescence signals can be monitored over extended periods (minutes) for long-term imaging of drug-induced non-neuronal cellular responses without causing cellular damage or fluorescence bleaching. *F*, photoreaction scheme and fluorescence-emission model of outward H^+^ pump rhodopsins. In AR3, fluorescence is emitted from the Q intermediate, which is generated by photon absorption from the precursor N intermediate. In AR3 variants such as QuasAr2 and Archon1, voltage-dependent fluorescence is proposed to originate from the excited state.

In addition to their application in controlling neuronal membrane potentials, several groups, including ours, have used outward and inward H^+^ pump rhodopsins to manipulate other cellular and physiological responses in animal cells (Fig. 5). Unlike the cytosol, cellular organelles have characteristic pH values (Fig. 1*B*) (2, 3). V-ATPases are known to acidify subcellular compartments in the secretory and endocytic pathways, such as synaptic vesicles, lysosomes, and endosomes (2, 3). Outward H^+^ pump rhodopsins such as AR3 and Arch-T have been targeted to synaptic vesicles and lysosomes, enabling optogenetic control of vesicular acidification. This manipulation leads to increased neurotransmitter accumulation in synaptic vesicles and alterations in hydrolase activity within lysosomes (Fig. 5*A*) (52, 53). An engineered inward H^+^ pump rhodopsin, the D217E mutant of Anabaena sensory rhodopsin (ASR), has been applied to early and late endosomes in hippocampal neurons and Purkinje cells (54). This approach enables light-dependent acidification or alkalization of the endosomal lumen, resulting in inhibition of AMPA receptor endocytosis and impaired motor learning in Purkinje cells.

As another optogenetic approach for manipulating cellular organelles, several H^+^ pump rhodopsins have been applied to mitochondria, where the PMF (−170 to −230 mV) across the inner membrane generated by respiration is essential for mitochondrial function (7). Multiple groups have heterologously expressed outward H^+^ pump rhodopsins in the mitochondrial inner membrane using mitochondrial targeting tags to optically manipulate the PMF in mammalian and insect cells as well as in the nematode *Caenorhabditis elegans* (Fig. 5*B*) (55, 56, 57, 58). This method generates PMF across the inner membrane, leading to suppression of respiration inhibition–induced cell death, enhanced ATP synthesis, lifespan extension in *C. elegans*, and reduced age-associated physiological decline. Collectively, these studies demonstrate that both outward and inward H^+^ pump rhodopsins can precisely manipulate pH and PMF within cellular compartments, allowing control of organelle-specific functions and elucidation of the relationship between pH/PMF and organelle physiology.

In contrast to the acidic compartments of cellular organelles, the cytosol is typically maintained at a neutral pH (∼7.2) under physiological conditions (2, 3, 59). Nevertheless, intracellular pH (*i*.*e*., cytosolic pH) can dynamically fluctuate (7.0–7.6), and such changes are thought to be associated with several cellular activities, including cell polarization, migration, differentiation, and cell homeostasis (59, 60, 61, 62). Donahue *et al*. applied Arch-T to retinal pigment epithelial cells and demonstrated that light-induced intracellular alkalization activates localized plasma membrane ruffling rather than inducing global cellular responses (63). To establish an optical method for controlling cell activities by modulating intracellular pH, cell death was selected as a target process based on the classical observation that human cultured cells immersed in alkaline solution (pH 9.0) undergo cell rounding, a characteristic morphological change associated with cell death, followed by subsequent cell death, strongly suggesting a link between intracellular alkalization and cell death (64, 65). First, HeLa cells expressing the outward and inward H^+^ pump rhodopsins AR3 and RmXeR were used to optically manipulate the cell death process in alkaline conditions through the control of intracellular pH (66). As expected, the rate of cell death increased upon AR3 expression but decreased upon RmXeR expression, reflecting their opposing abilities to enhance or suppress intracellular alkalization in the surrounding alkaline environment. Intracellular alkalization induced by AR3 was also shown to trigger mitochondrial-mediated apoptotic cell death under physiological, neutral extracellular pH in several mammalian cell lines, including cancer model cell lines such as MC38 and B16F10 (66, 67) (Fig. 5*C*). Moreover, we demonstrated that AR3-induced cell death can be applied to cancer cells in living mice, suggesting its potential utility in the development of novel light-based cancer therapies (67). Because light irradiation of AR3 and RmXeR can bidirectionally alter intracellular pH by at least 1.1 units (66), we anticipate that such optical methods will serve as powerful tools for investigating the relationships between intracellular pH and diverse cellular activities in animals.

In addition to the ectopic expression of rhodopsins in animal cells, the delivery of rhodopsin-containing, light-responsive molecular systems into cells offers another strategy for optically controlling cellular responses. Liposomes have long been used in rhodopsin research to examine the biophysical properties of these proteins within membrane environments and to evaluate the ion-transport activities of ion-transporting rhodopsins. Furthermore, liposomes are widely employed as biological carriers for delivering chemical and biological compounds, including therapeutic agents, into cells and tissues (68). Building on these applications, light-disruptive, rhodopsin-based liposomes were developed to release encapsulated substrates upon illumination (Fig. 5*D*). In this approach, the inward H^+^ pump rhodopsin RmXeR was reconstituted into liposomes composed of the pH-sensitive lipids 1,2-dioleoyl-sn-glycero-3-phosphoethanolamine (DOPE) and cholesteryl hemisuccinate (CHEMS) (69). Illumination triggered the release of encapsulated molecules, including the fluorescent dye calcein and mRNA encoding enhanced green fluorescent protein, into HeLa cells (Fig. 5*D*). The released mRNA was further shown to be translated, resulting in expression of the encoded protein. Although this approach does not constitute a classical optogenetic technique, it provides a new modality for light-controlled intracellular delivery of biomolecules.

## Optical imaging of membrane potentials using H^+^ pump rhodopsins and their variants

In addition to their use as optical tools for controlling biological activities, H^+^ pump rhodopsins and their variants have also been developed as optical reporters of membrane potential, known as genetically encoded voltage indicators (GEVIs) (70, 71). Dr Cohen’s group first demonstrated that the outward H^+^ pump rhodopsin PR exhibits voltage-dependent near-infrared fluorescence changes in *E*. *coli* (72). They subsequently applied AR3 to animal neurons and successfully imaged neuronal activity by detecting membrane voltage changes with high spatiotemporal resolution (Fig. 5*E*) (73). Since then, numerous engineered AR3 variants have been generated through random mutagenesis to improve fluorescence yield, signal-to-noise ratio, membrane localization, and response kinetics (74, 75, 76, 77, 78). In general, AR3 and its variants are used as GEVIs under intense laser illumination (1–1000 W/cm^2^) to detect the intrinsically dim fluorescence emitted by rhodopsins (Fig. 5*E*). However, such strong illumination can induce severe cellular damage, particularly during long-term imaging over several minutes. Voltage-sensitive fluorescence from AR3 and Archon1 has been successfully detected in various mammalian cell lines using low-intensity LED illumination (0.15 W/cm^2^) (79) (Fig. 5*E*). This system enables real-time imaging of drug-induced, slow membrane potential changes over extended periods without causing cellular damage or fluorescence bleaching (Fig. 5*E*). These results indicate that rhodopsin-based GEVIs are applicable to real-time optical imaging of both neural and non-neural cellular responses. Spectroscopic analyses have suggested that the near-infrared fluorescence of AR3 originates from the Q intermediate formed upon photon absorption from the preceding N intermediate, and that fluorescence intensity is strongly dependent on membrane voltage (80) (Fig. 5*F*). In contrast, variants such as QuasAr2 and Archon1 emit fluorescence directly from their excited states (Fig. 5*F*) (81). In addition, several other microbial rhodopsins, including Anabaena sensory rhodopsin (ASR), halorhodopsin from *Natronomonas pharaonis* (NpHR), and outward H^+^ pump rhodopsins such as GR and bacteriorhodopsin (HwBR) from *Halobacterium walsbyi*, were found to exhibit strong near-infrared fluorescence (82, 83). We anticipate that not only outward H^+^ pump rhodopsins but also other classes of rhodopsins will serve as powerful GEVIs for optogenetic imaging of membrane potential dynamics in both neural and non-neural cells.

## Future investigations of optogenetics using H^+^ pump rhodopsins and beyond

### Enhancement of H^+^ pump activity by secondary chromophores

In this review, we have described optogenetic applications of H^+^ pump rhodopsins for manipulating physiological activities in both animal and bacterial cells (Table 1). Accumulated studies indicate that H^+^ pump rhodopsins provide a unique means to non-invasively and precisely manipulate cellular pH and PMF without directly perturbing endogenous H^+^ transport systems, such as by gene knockdown, knockout, overexpression, or pharmacological activation or inhibition (51, 63, 66). Therefore, further expansion of the molecular toolkit of H^+^ pump rhodopsins, together with continued advances in genetic technologies, will enable increasingly fine-tuned control of intracellular pH dynamics and PMF. Xanthorhodopsin (XR) from the extreme halophile *Salinibacter ruber* was reported as the first carotenoid-binding rhodopsin (84), and subsequently studies have revealed that several H^+^ pump rhodopsins, including PRs from flavobacteria and other XRs, can bind carotenoid antenna molecules as secondary chromophores (85, 86, 87). This carotenoid-binding feature extends their spectral sensitivity and enhances their light-harvesting efficiency for H^+^ pump activity. Thus, the exploitation and engineering of novel carotenoid-binding H^+^ pump rhodopsins are expected to further expand the versatility of optical pH and PMF control, by enabling enhanced light-harvesting efficiency and broader spectral tunability under diverse biological and physicochemical conditions.

### Advanced optogenetic applications of H^+^ pump rhodopsins

**Figure 6:**
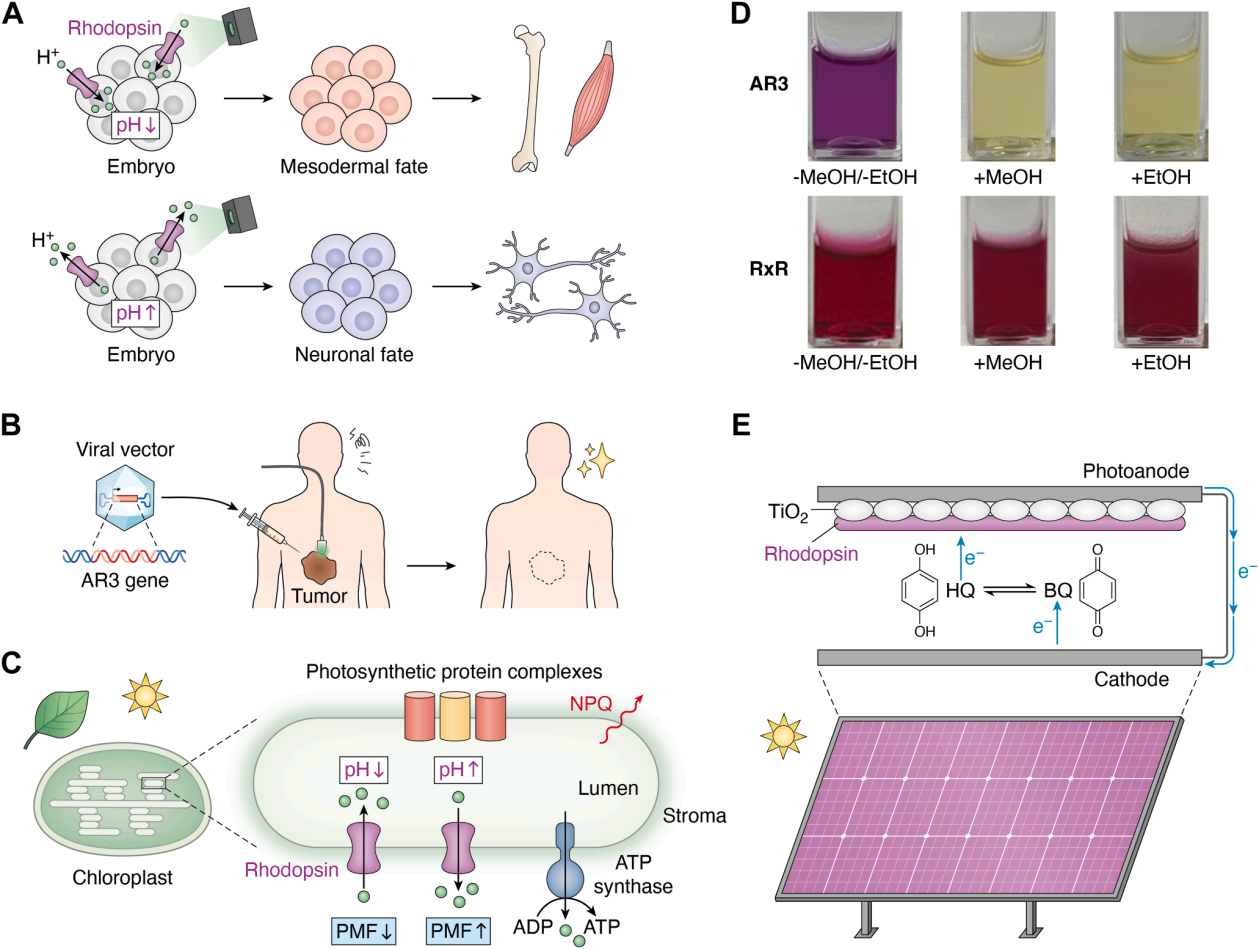
**Future investigations of applications of H^+^ pump rhodopsins.***A*, optical control of cell differentiation through modulating intracellular pH during development. *B*, potential application to cancer therapy through light-induced cell death in tumors. *C*, regulation of photosynthetic activity in plants through control of the thylakoid PMF. *D*, methanol and ethanol resistance of AR3 and RxR. Photographs of purified AR3 and RxR samples in buffer (50 mM Tris–HCl, 0.05% (w/v) n-dodecyl-β-D-maltoside, pH 7.0) in the presence or absence of 40% (v/v) methanol or ethanol. *E*, conceptual application of H^+^ pump rhodopsins in biohybrid photovoltaic devices, in which light-induced electron transfer generates photocurrents for solar energy conversion. In these systems, the HQ/BQ redox matrix regenerates photooxidized rhodopsins through continuous electron donation.

Recent advances in genetic engineering now allow precise regulation of ectopic gene expression in specific cell types and subcellular compartments across diverse developmental and physiological stages. Notably, several studies have demonstrated that intracellular pH plays critical roles in tissue development and metabolism through the regulation of intracellular signaling pathways, such as WNT and mTORC1 signaling, in animal cells (60, 88, 89). In this context, emerging optogenetic applications of H^+^ pump rhodopsins are expected to enable light-dependent control of developmental processes (*e*.*g*., differentiation, tissue growth, and morphogenesis) as well as metabolic functions (*e*.*g*., glycolysis, the citric acid cycle, oncogenesis, and tumor progression), thereby providing new opportunities to elucidate their underlying molecular mechanisms (Fig. 6*A*). More recently, optogenetic approaches have begun to be developed as therapeutic tools, with a representative example being vision restoration through viral vector–mediated delivery of rhodopsin genes to target retinal cells (90). Given our demonstration of the high potential of light-induced cell death using AR3 in mouse cancer models (67) (Fig. 5*C*), future validation under clinically relevant tumor conditions in humans will be essential for developing therapeutic strategies based on this approach (Fig. 6*B*). Collectively, these ongoing and emerging applications highlight the broad potential of H^+^ pump rhodopsins for probing cellular physiology and for designing next-generation optogenetic and therapeutic strategies.

While optogenetic tools have been progressively developed for manipulating cellular functions in animal and bacterial cells, much less progress has been made in plant systems. Although rhodopsin genes have been identified in animals and bacteria, no rhodopsin genes have been reported in plants. Moreover, plants are thought to lack retinal, the chromophore essential for rhodopsin function, although they can synthesize carotene, a precursor of retinal. These limitations have hindered the optogenetic application of rhodopsins in plant cells. As the first demonstration of plant optogenetics, it was reported that the introduction of cation channel rhodopsin–induced membrane depolarization by adding endogenous retinal, implying the optogenetic potential of rhodopsins (91). After that it was reported that the introduction of GtACR1 together with genes required for retinal biosynthesis induced membrane depolarization through large anion currents, enabling non-invasive manipulation of plant growth and leaf development (92). Since then, several optogenetic applications using ACRs and CCRs have been reported; however, there have been no reports introducing H^+^ pump rhodopsins into plant cells (92, 93, 94). Because proton flux across the plant plasma membrane and the thylakoid membranes in chloroplast is involved in various cellular and physiological processes, including photosynthesis, non-photochemical quenching (NPQ), stomatal movement, and signal transduction (95, 96, 97), the application of H^+^ pump rhodopsins to plant cells, including chloroplasts, is expected to open a new era of optogenetic control of plant physiology (Fig. 6*C*).

### Applications of H^+^ pump rhodopsins in chemical reactions

Beyond the optical control of biological activities, H^+^ pump rhodopsins have considerable potential to drive a wide range of chemical reactions, including applications in biofuel production, artificial photosynthesis, and solar energy conversion. As described above, outward H^+^ pump rhodopsins have been reported to induce ATP synthesis and promote the production of valuable chemical compounds by modulating carbon metabolism in bacteria and artificial liposome systems (43, 44, 45, 98) (Fig. 4*A*). These findings enable the construction of artificial photosynthetic systems linked to carbon metabolism in engineered bacteria and artificial liposomes, with potential applications in biofuel production and artificial photosynthesis. In addition, several studies have demonstrated photovoltaic devices composed of purple membranes expressing bacteriorhodopsin (HsBR) combined with conductive oxide electrodes, such as SnO_2_, TiO_2_, and indium–tin oxide (ITO) (99, 100, 101, 102). In these systems, photoactivation of HsBR induces local pH changes near the electrode surface and promotes electron transfer from HsBR to the electrode surface, which generate transient photocurrents. To achieve continuous rather than transient power generation, recent biohybrid solar cells utilize HsBR as a photosensitizer integrated with a hydroquinone/benzoquinone (HQ/BQ) redox electrolyte (102). In this system, the light energy captured by the retinal chromophore, which biologically drives proton pumping, is redirected toward direct electron injection into the TiO_2_ conduction band. The photooxidized HsBR is continuously regenerated through electron donation from the HQ/BQ redox matrix. These findings demonstrate that the light-harvesting properties of proton-pumping rhodopsins can be utilized not only for proton transport but also for steady-state electrochemical charge separation in photovoltaic devices. Such studies propose that incorporating H^+^ pump rhodopsins into artificial semipermeable membranes or lipid layers can lead to novel systems for artificial photosynthesis and solar cells. For these surface-based applications, it is essential that rhodopsins exhibit high stability under harsh experimental conditions, including exposure to organic solvents used to process electrode and membrane materials. Several rhodopsins from thermophilic bacteria, including Thermophilic rhodopsin (TR), *Rubrobacter xylanophilus* rhodopsin (RxR), and *Bellilinea* Na^+^ pump rhodopsin (BeNaR), have been identified and shown to possess high thermal stability (103, 104, 105, 106). To further examine the stability of the outward H^+^ pump rhodopsin RxR in organic solvents, its visible absorption properties were monitored in standard buffer containing 40% (v/v) methanol or ethanol and compared them with those of the conventional H^+^ pump rhodopsin AR3 (Fig. 6*D*), based on previous reports that proteins from thermophilic bacteria often exhibit enhanced resistance to organic solvents. While the characteristic red coloration of AR3 was lost and a yellow coloration, due to the release of retinal from denatured AR3, was observed in methanol- or ethanol-containing solution, RxR retained its visible color under the same conditions, clearly indicating its high resistance to organic solvents such as methanol and ethanol. These results suggest that thermally and chemically stable H^+^ pump rhodopsins represent promising candidates for incorporation into photovoltaic devices and related artificial energy-conversion systems (Fig. 6*E*).

### Current limitations and challenges in optogenetic applications using H^+^ pump rhodopsins

Intracellular pH has traditionally been manipulated using chemical approaches, such as protonophores and inhibitors of endogenous H^+^ pumps (2, 107, 108). However, these methods can induce artificial effects through perturbation of endogenous cellular functions, offer limited temporal control, and are generally irreversible. In contrast, optogenetic approaches using H^+^ pump rhodopsins enable reversible and minimally invasive control of cytoplasmic and subcellular pH with high spatial and temporal resolution. A major limitation is that most outward and inward H^+^ pump rhodopsins absorb green light, which exhibits limited penetration into deep tissues. Therefore, the development of red- or near-infrared-absorbing H^+^ pump rhodopsins through protein engineering, as well as the discovery of new natural variants, will be important for expanding *in vivo* applications.

## Conclusion

We have introduced and highlighted the high potential of rhodopsins, particularly H^+^ pump rhodopsins, as optogenetic tools from both biological and physicochemical perspectives. Optogenetic applications of H^+^ pump rhodopsins have been progressively developed for manipulating a wide range of physiological activities in animal and bacterial cells, and are expected to be extended to broader biological contexts, including plant physiology and next-generation therapeutic strategies. Based on these accumulated studies, we envision emerging directions that extend beyond classical optogenetics toward the optical control of bioenergetic and chemical processes.

## Data availability

All data generated or analyzed during this study are included in this published article and its supporting information files. Raw data are available upon request

## Conflict of interest

The authors declare that they have no conflicts of interest with the contents of this article.
